# Identification of Co-Existing Mutations and Gene Expression Trends Associated With K13-Mediated Artemisinin Resistance in *Plasmodium falciparum*

**DOI:** 10.3389/fgene.2022.824483

**Published:** 2022-04-06

**Authors:** Mukul Rawat, Abhishek Kanyal, Deepak Choubey, Bhagyashree Deshmukh, Rashim Malhotra, DV Mamatharani, Anjani Gopal Rao, Krishanpal Karmodiya

**Affiliations:** ^1^ Department of Biology, Indian Institute of Science Education and Research, Pune, India; ^2^ Life Science Research Unit, Persistent Systems Limited, Pune, India

**Keywords:** malaria, *Plasmodium falciparum*, artemisinin resistance, Kelch13 mutations, genomics, transcriptomics

## Abstract

*Plasmodium falciparum* infects millions and kills thousands of people annually the world over. With the emergence of artemisinin and/or multidrug resistant strains of the pathogen, it has become even more challenging to control and eliminate the disease. Multiomics studies of the parasite have started to provide a glimpse into the confounding genetics and mechanisms of artemisinin resistance and identified mutations in Kelch13 (K13) as a molecular marker of resistance. Over the years, thousands of genomes and transcriptomes of artemisinin-resistant/sensitive isolates have been documented, supplementing the search for new genes/pathways to target artemisinin-resistant isolates. This meta-analysis seeks to recap the genetic landscape and the transcriptional deregulation that demarcate artemisinin resistance in the field. To explore the genetic territory of artemisinin resistance, we use genomic single-nucleotide polymorphism (SNP) datasets from 2,517 isolates from 15 countries from the MalariaGEN Network (The Pf3K project, pilot data release 4, 2015) to dissect the prevalence, geographical distribution, and co-existing patterns of genetic markers associated with/enabling artemisinin resistance. We have identified several mutations which co-exist with the established markers of artemisinin resistance. Interestingly, K13-resistant parasites harbor α-ß hydrolase and putative HECT domain–containing protein genes with the maximum number of SNPs. We have also explored the multiple, publicly available transcriptomic datasets to identify genes from key biological pathways whose consistent deregulation may be contributing to the biology of resistant parasites. Surprisingly, glycolytic and pentose phosphate pathways were consistently downregulated in artemisinin-resistant parasites. Thus, this meta-analysis highlights the genetic and transcriptomic features of resistant parasites to propel further exploratory studies in the community to tackle artemisinin resistance.

## Introduction

Malaria, a disease caused by a unicellular parasite belonging to the genus *Plasmodium*, has plagued mankind since times immemorial. *Plasmodium falciparum* is perhaps the most virulent species of the genus and is also associated with the more morbid and often lethal manifestations of malaria. The World Health Organization (WHO), which keeps a close tab on the global prevalence of the disease, reported an estimated 229 million cases of malaria and 409,000 deaths in 2019 alone (World Health Organization, 2020). Clinical observations in the field and *in vitro* studies of drug pressure regimens have shown the parasite’s ability to evolve drug resistance rapidly (Bakhiet et al., 2019; Uwimana et al., 2020). WHO recommended use of artemisinin-based combination therapies (ACTs) in 2001. However, several studies have shown the selection of mutations associated with resistance to multiple drugs in *P. falciparum*, leading to the Accelerated Resistance to Multiple Drug phenotype (Rathod et al., 1997; Le Bras et al., 2003).

Artemisinin is a sesquiterpene lactone derived from the wormwood plant *Artemisia annua*. It is highly potent in reducing’ the asexual stage parasite load in infected humans with no major side-effects. Owing to its short plasma half-life, it is often coupled with other longer lasting compounds in an ACT to prevent the relapse of parasites leading to emergence of resistance. The drug carries an endoperoxide bridge that is cleaved in the presence of free Fe^2+^ ions in the parasite, generated by digestion of hemoglobin in the food vacuole. Upon activation the drug goes into a free radical cascade that forms adduct with and damages biomolecules in the parasite cell. Recent click chemistry–based experiments designed to tag and identify molecular interactions of artemisinin in cells have identified factors involved in chaperoning, cellular transport, nucleic acid synthesis, and antigenic variation (Ismail et al., 2016a). Clinical resistance to artemisinin was first reported by Arjen Dondorp et al. in 2009 (Dondorp et al., 2009). It was identified as a delay in the clearance of parasite following treatment with artemisinin. Furthermore, to confound a clear definition of artemisinin resistance, *in vitro* drug sensitivity studies were shown to have little or no changes in artemisinin IC_50_ values even in field isolates with delayed clinical clearance times. Hence, a new standard of artemisinin sensitivity assay was developed called the ring-stage survival assay (RSA), which mimics the pharmacokinetic properties of the drug, including peak plasma concentrations and exposure cycles normally observed for artemisinin in patients (Witkowski et al., 2013). A survival of >1% of parasites in the RSA deems them resistant to artemisinin in *in vitro* settings. *In vitro* studies established that artemisinin resistance is associated primarily with the ring stage of *Plasmodium* life cycle; it is considered as “partial resistance” due to its stage specific nature. Artemisinin-resistant parasites, i.e., those with delayed clearance phenotype, result from the ability of the ring stage to enter into a “dormant state” induced by artemisinin stress. Parasites stay in a dormant stage for different duration depending on the genetic back ground (Teuscher et al., 2010).

**FIGURE 1:**
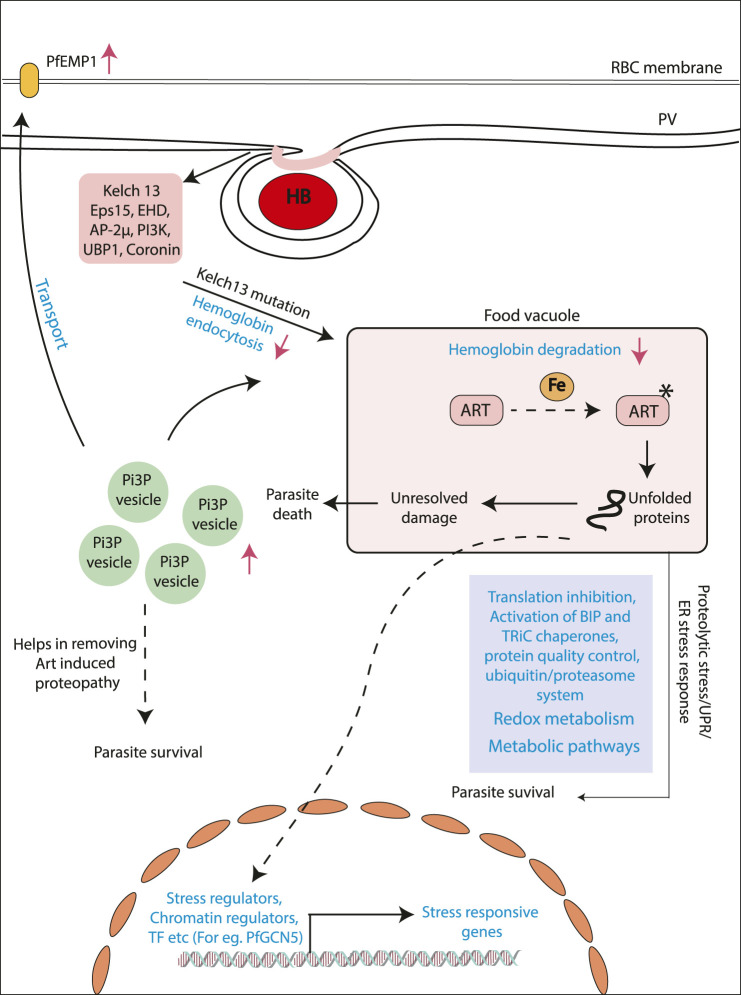
Mechanisms proposed for artemisinin resistance in *P. falciparum*. Model showing the different mechanism proposed for artemisinin resistance generation. Artemisinin treatment results alkylation of several proteins resulting in the state of stress within the parasites. Different pathways like unfolded protein response and ubiquitin/proteasome system. Stress-like state results in the upregulation of stress induced genes by transcriptional regulators (e.g., PfGCN5). Another mechanism addresses the role of upregulated PI3P levels in the artemisinin-resistant parasites. Increased level of this lipid results in increased PI3P vesicles, which houses various proteins helps in the removing artemisinin induced proteopathy. Recent studies have identified decreased hemoglobin uptake and degradation. This ultimately results in decreased artemisinin activation and, hence, decreased artemisinin sensitivity.

Mutations in the PfKelch13 (K13) gene were identified to be definitively associated with artemisinin resistance (Ariey et al., 2014). One of the first studies to identify the role of K13 mutations in artemisinin resistance showed the enhancement of PI3P enriched vesicles carrying proteostatic factors in K13 mutant artemisinin resistance parasites (Figure 1) (Bhattacharjee et al., 2018). Some of the very recent studies identify K13-enriched endosomal compartments that directly regulate the endocytosis of hemoglobin into the host cell (Birnbaum et al., 2020). Mutations in K13 or its associated partner proteins in this vacuolar compartment tend to reduce the endocytosis of hemoglobin into parasite cells resulting in reduced drug activation (Figure 1) (Birnbaum et al., 2020). K13 mutations may, however, be predominant only in a subset of global populations, especially from Southeast Asia (SEA) (MalariaGEN Plasmodium falciparum Community Project, 2016). Although reports of artemisinin resistance from other parts of the world are gradually emerging, a strong correlation with K13 mutations is not observed. Subsequent independent drug adaptation studies have identified numerous mutational markers associated with artemisinin resistance *in vitro* (Wang et al., 2020). As such, there may be no one “universal identifier” of artemisinin resistance, but a number of them each specifically built/selected upon a complicated genetic background shaped by years of differential evolution in the field (Wilairat et al., 2016).

Furthermore, considering the sheer abundance of cellular targets for artemisinin in the cell, it is plausible to assume that modes of developing resistance must be prolific as well. An insight into the possible systemic mechanisms for artemisinin resistance has come from population transcriptomic studies (Mok et al., 2015). The parasite shows distinct gene expression changes, allowing for delayed progression of the ring stage of parasite, where it is transcriptionally least active and presents very limited proteome for damage (Mok et al., 2015). The parasite then seems to subsequently enter into a state of heightened stress response by enhancing the production of stress response factors pertaining to reactive oxygen stress complex, T-complex protein 1 ring complex, endoplasmic reticulum–resident unfolded protein response (UPR) pathway, and the ER-associated degradation (Mok et al., 2015). Studies have also linked the activated UPR in the endoplasmic reticulum to a downstream translational arrest (Figure 1) (Mok et al., 2015; Suresh and Haldar, 2018). The transcriptional profiles unique to resistant parasites are believed to be overlaid on a complex interaction of environmental pressures and selective evolution of genotypes (Mok et al., 2011; Dwivedi et al., 2017). Epigenetic factors that can respond to environmental perturbations in real time and evoke rapid responses in parasites may also contribute to resistance as shown in a recent study (Rawat et al., 2021). Identifying the markers and mechanisms of resistance in K13-independent resistance is also the next challenge and comparative multiomics can bolster investigations into these questions. Artemisinin combination therapy while highly effective and less susceptible to complete failure is nonetheless prone to gradual failure by accumulation of progressive single-nucleotide polymorphisms (SNPs) mediating resistance to not only artemisinin but also the partner drug (Wang et al., 2016). Several SNPs have been profiled in association with failure of the partner drugs currently employed in the ACT alongside artemisinin (Antony et al., 2016; Jiang et al., 2021). Few such examples are lumefantrine (mutations in the chloroquine resistance transporter, Pfcrt K76T, and multidrug resistance gene Pfmdr1 N86Y), amodiaquine (mutations in the Pfcrt K76T and Pfmdr1 N86Y), piperaquine (copy number variations in plasmepsin 2-3), and sulfadoxine-piperaquine dihydrofolate reductase gene pfdhfr (51I, 59R, and 108N), and the double mutant in dihydrofolate pteroate synthase gene pfdhps (437G and 540E) have been reported to be rendered useless by mutations (Slater et al., 2016).

With the ever-increasing usage of ACT worldwide, it becomes especially important to screen isolates from the field and perform genotype–phenotype screenings to assess the efficacy of drugs currently employed in the field. The markers emerging out of these molecular epidemiology studies also need validation by *in vitro* studies that aim at observing the effects to engineering these mutations on parasite phenotypes (Stokes et al., 2021). Such studies highlight the need of system-wide analysis, characterization, and validation of resistance marker data.

The purpose of this analysis cum review is to revisit the ample genomic and transcriptional datasets described for artemisinin resistance in the Greater Mekong Subregion in SEA and Africa. We aim to summarize our current understanding of the genotypic landscape and highlight the transcriptomic trends in artemisinin-resistant parasites. Our focus has been to summarize the genetic markers associated with artemisinin resistance and their geographical prevalence and to investigate the patterns of their co-existence in isolates reported with artemisinin resistance. Initial transcriptome studies have provided us valuable insights into the possible physiological adaptations of the parasites to artemisinin and highlighting key factors from these datasets may fuel future investigations. In our review of the transcriptomic dataset, we aim at identifying specific factors that show consistent deregulation in association with artemisinin resistance. These highlighted factors can be further investigated for their role in emergence of drug resistance and be potentially considered for lead candidates for targeting in ongoing/future pharmacological intervention strategies on artemisinin resistance. With this review, we also aim to shift focus of investigations from broad biological pathways to specific factors that may have important roles to play in artemisinin drug resistance generation and sustenance.

## Materials and Methods

### Whole Genome Sequencing Data Access and Analysis

SNP data were downloaded from Pf3K (Pilot data release 4) MalariaGEN (The Pf3K project, 2015) to analyze key mutations present in the PfKelch13 gene (PF3D7_1343700) (located on Chromosome number 13) (MalariaGEN Plasmodium falciparum Community Project, 2016). Genomes of 2517 isolates were available from 15 different geographical locations, majorly categorized into Africa (1,501) and Asia (1,010) subcontinents and some lab strains (6). Variant annotation for the data was done using snpEff version 4.3 (Cingolani et al., 2012). Fourteen of the K13 SNPs known to be associated with artemisinin resistance were assessed for their prevalence across different geographical region and co-existence among the 424 isolates they were found in.

Four SNPs from chloroquine resistance transporter Pfcrt gene (K76T, A220S, I356T, and R371I) and five SNPs for multidrug resistance Pfmdr gene (N86Y, E130K, Y184F, S1043C, and N1042D) were analyzed for co-existence with three definitive K13 markers of artemisinin resistance (C580Y, R539T, and I543T). Further exploration of background genomic variants co-existing with the K13 mutations in artemisinin resistance was identified using the 359 samples, which show one of the three major K13 mutations (C580Y, R539T, and Y493H). For an SNP to be filtered for co-existence with K13 mutations it had to be present in at least 75% of K13 mutant isolates (2 sigma value is 81.27%, covering more than 95% of the population). We performed a chi-squared test by comparing the combination wild-type (WT)/mutant instances of K13 with WT/mutant instances of the candidate genes in a 2 × 2 grid format. We applied the chi-squared independence test with a *p*-value cutoff of 0.05 and degree of freedom 1 (standard for a 2 × 2 grid) (Supplementary Table S1). In-house Perl and R scripts were used to analyze the data for co-existence. Variant annotation was done for all the SNPs using the tool snpEFF and only those SNPs that were non-synonymous mutation were considered for further analysis (Cingolani et al., 2012). Plots were generated using R and GraphPad (Swift and sciences, 1997; Tippmann, 2015).

### Transcriptome Data Access and Review

We reviewed transcriptomic datasets from three studies with the aim to identify genes that were deregulated consistently across multiple studies. Gene list for the different protocol was drafted from the previously published literature as well as using PlasmoDB Malaria Parasite Metabolic Pathway (MPMP) database. The studies of Mok et al. and Rocomora et al. were used to identify genes that were deregulated (Mok et al., 2015; Mok et al., 2011; Rocamora et al., 2018). The studies of Mok et al. (2011 and 2015) are the *ex vivo* transcriptomic dataset, whereas Rocamora 2018 study used *in vitro–*selected artemisinin-resistant isolates (Chan and Walmsley, 1997). We chose to extract the list of genes mentioned as significantly deregulated in the respective studies as per the statistical criteria set by the original authors and as described in Supplementary Table S2. For this review, we considered genes mentioned in these studies with at least two-fold differential expression. Those that showed consistent deregulation across at least two of the three independent studies were shortlisted. The aim of this transcriptomic review is to identify genes (relevant to important biological pathways contributing to drug resistance), showing consistent deregulation.

## Results and Discussion

### Genomic Landscape of Artemisinin Resistance: Geographical Prevalence and Trends of Co-Existence

**FIGURE 2:**
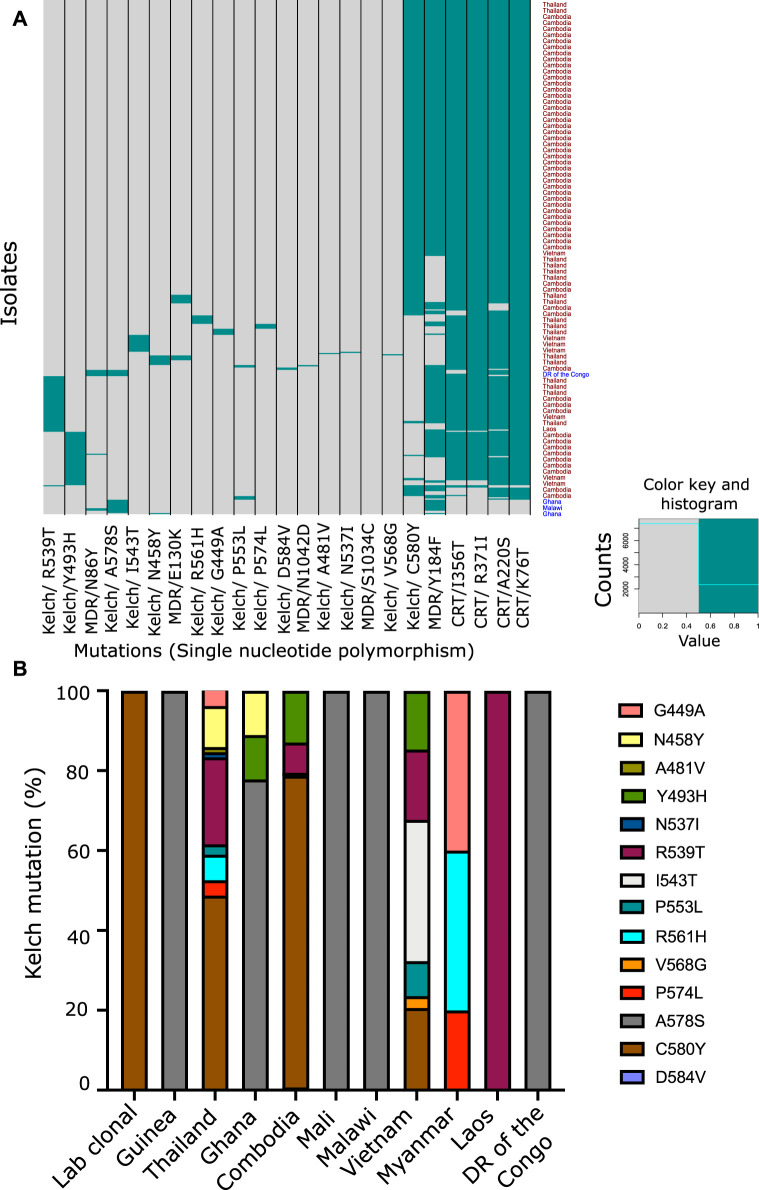
Co-prevalence of the PfKelch13 mutations with other markers of multidrug resistance. **(A)** SNP data were downloaded from Pf3K (Pilot data release 4) MalariaGEN to analyze key mutations present in the K13 gene (PF3D7_1343700). Genomes of 2,517 isolates were available from 15 different geographical locations, majorly categorized into Africa (1,501) and Asia (1,010) subcontinents and some lab strains (6). Variant annotation for the data was done using snpEff version 4.3. Heatmap representing the mutation present in the different isolates from different geographical areas. Pfcrt (Chloroquine Resistance Transporter) and Pfmdr (Multidrug Resistance Transporter) mutations were also plotted along with the known K13 mutations to understand the co-prevalence of these mutations. **(B)** Fourteen of the Kelch SNPs known to be associated with artemisinin resistance were assessed for their prevalence across different geographical region and co-existence among the 424 isolates they were found in. Percentage proportion of different K13 mutation prevalent in the different countries used for the study.

To explore the genetic landscape relevant to artemisinin and multidrug resistance, we focused on the validated genetic markers associated strongly with drug resistance across the world. We laid special emphasis on the K13, the chloroquine resistance transporter (Pfcrt) and the multidrug resistance (Pfmdr) SNPs to identify their 1) abundance, 2) co-existence in isolates, and 3) geographical prevalence. We downloaded the Pf3K SNP dataset for 2,517 isolates from 15 countries in the SEA, South Asia, and Africa and six lab strains of the parasite. To have a better understanding of the genotype associated with artemisinin resistance, we focused on the isolates harboring any of the 14 K13 mutations confirmed to be correlated with artemisinin resistance in GWAS (Supplementary Table S3) (Miotto et al., 2015). Among these, the C580Y SNP was found to be the most abundant, followed by R539T and Y493H (Figure 2A and Supplementary Table S3). Most of the other SNPs were at very low frequency as summarized in the (Figure 2A and Supplementary Table S3). It is speculated that mutations in the K13 protein result in either depletion of function or destabilization of the protein itself (Ariey et al., 2014; Coppée et al., 2019). A dysfunctional, mutant K13 or even lower amounts of WT K13 have been linked to impediments in the hemoglobin endocytosis leading to a subsequently lower activation of artemisinin as a result (Birnbaum et al., 2020). Although this protects the parasites from the damage caused by the drug, it infers a cost of poor growth and proliferation defects due to lack of amino acids obtained from hemoglobin catabolism. This explains why C580Y, which has low resistance to offer in RSA (∼8% survival in RSA), is far more abundantly spread across SEA as compared to the other mutations (R539T or I543T) that offer more survival under drug pressure but may cause proliferation bottlenecks due to limited nutrient availability (Ariey et al., 2014; Nair et al., 2018; Tirrell et al., 2019; Birnbaum et al., 2020). Furthermore, we looked for co-existence of the K13 SNPs with other drug resistance markers, if any. Although mutations in the markers for chloroquine resistance (Pfcrt and Pfmdr) and sulfadoxine and pyrimethamine (Pfdhfr/Pfdhps) can often co-exist, the same does not hold true for mutations of the K13 gene (Figure 2A). We observed prevalence of only one variant of K13 at a time in the isolates. This may well be because harboring even two individually destabilizing mutations in the core K13 protein may amplify the overall instability of the protein, thus resulting in non-viability of the parasites. Interestingly, from SEA, Cambodia reported three isolates with co-existence of C580Y/Y493H K13 SNPs; two isolates with C580Y and one isolate from Thailand reported a double mutation (C580Y + R539T) (Figure 2A and Supplementary Table S3). However, presence of multiple K13 mutations can also be result of multiclonal infections. Among the SEA countries alone, the most prominent K13 mutations stood as C580Y, R539T, Y493H, and I543T in the decreasing order (Figure 2B and Supplementary Table S3). The countries in Africa reported scarcity of K13 mutations with A578S being the only predominant variant (Figure 2B and Supplementary Table S3). The regionally distinct enrichment of K13 mutations between African and Southeast Asian countries is striking.

#### Inspection of Genetic Backgrounds Against Which Specific K13 SNPs are Co-Existed

We proceeded to analyze the co-existence of K13 polymorphisms with markers associated with resistance to drugs that have been previously employed in the field and have subsequently been taken out of active usage. Pfcrt and Pfmdr SNPs were assessed for chloroquine resistance (Supplementary Table S3) (Venkatesan et al., 2014). We prepared a matrix with binary representation (0 for absent; 1 for present) for the prevalence of various SNPs in isolates from across the world. From this, we filtered out the isolates for distinct K13 mutations and calculated the percentage of isolates that reported co-existing mutations in the MDR or CRT locus. CQ resistance SNPs are among the most prevalent in the field, owing to rampant uncontrolled usage of CQ in the past decades, which led to widespread and rapid development of resistance that spread across the world. It is believed that these SNPs may form a genotypic background, which stabilizes the selection of K13 SNPs (Miotto et al., 2015). We observed a trend of co-existence of K13, Pfcrt, and Pfmdr mutations among the isolates (Table 1 **)**. A majority of the Pfcrt mutations were prevalent in the background of the K13 genotype and might be even permissive for establishment of K13, whereas only one Pfmdr mutation (Y184F) was found to share its existence with the K13 SNPs (Figure 2A, Table 1 and Supplementary Table S3). When we sorted the samples to filter out for the prominent Pfcrt SNPs, we found enrichment of isolates from African countries. These isolates were enriched for the K13 A578S (Supplementary Table S3). It is plausible that the decade-long withdrawal of chloroquine from the African subcontinent may have diminished the abundance of popular Pfcrt mutations in the region. It is also plausible that A578S K13 mutations are more easily selected for in this unique genetic background (marked by absence of the popular Pfcrt markers).

**TABLE 1:** Co-prevalence of the K13 mutations with other markers of multidrug resistance.

K13 genotype	CRT genotype (% positive)				MDR genotype (% positive)				
	K76T	A220S	I356T	R371I	N86Y	E130K	Y184F	S1034C	N1042D
C580Y	99.2	97	96.3	97.4	0	2.5	83.5	0	0
Y493H	100	98	91	91	2	0	52	0	0
R539T	98	96	98	98	0	0	90	0	0

#### Genomic Variants Co-Existing With the Artemisinin Resistance Markers

**FIGURE 3:**
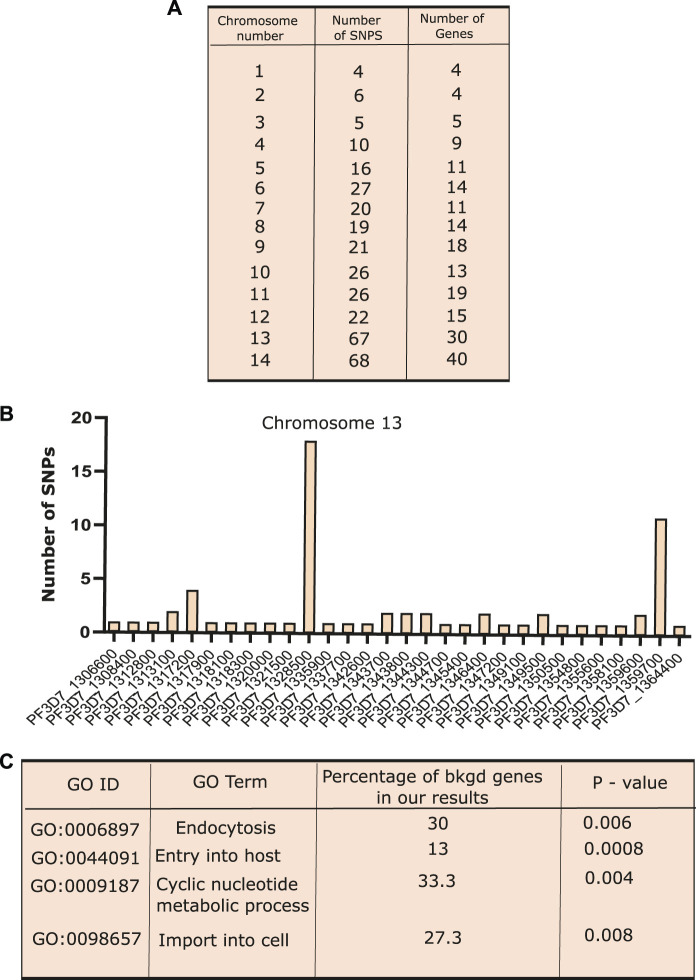
Co-existence of different SNPs along with PfKelch13 mutations. **(A)** Exploration of background genomic variants co-existing with the K13 mutations in artemisinin resistance was identified using the 359 samples, which show one of the three prime K13 mutations (C580Y, R539T, and Y493H). In order for an SNP to be filtered for co-existence with K13 mutations, it had to be present in at least 75% of K13 mutant isolates. Variant annotation was done for all the SNPs using the tool snpEFF and only those SNPs that were non-synonymous mutation were considered for further analysis. Table presenting the number of SNPs identified present in the 75% of the K13 mutants isolates and present in less than 25% of the sensitive (K13 mutant absent) isolates in various chromosomes. **(B)** Bar graph showing the number of SNPs present on the different genes over chromosome 13. **(C)** Gene ontology performed for the genes showing SNP co-existing with K13 mutations. Different biological processes like “endocytosis”, “locomotion”, “cell division”, and “response to drug” were found to be enriched. Plots were generated using R and GraphPad.

To detect the genomic variants that co-existed along with K13 mutations in artemisinin-resistant isolates, we screened the background genotype of the three major K13 mutants (C580Y, Y493H, and R539T). The SNP data were downloaded from MalariaGEN Network (The Pf3K project, pilot data release 5, 2016) repositories for all the *P. falciparum* chromosomes (MalariaGEN Plasmodium falciparum Community Project, 2016). A total of 359 isolates carried one of the three major K13 SNPs and flagged as “resistant”, whereas the rest of the isolates lacking definitive K13 mutations were marked as “sensitive”. Variant annotation was done using the tool snpEFF, and the non-synonymous SNPs were considered for further assessment (Cingolani et al., 2012). To consider an SNP as co-existing alongside K13 mutation, we set a cutoff criterion of co-existence in at least 75% resistant isolates (269 genomes) and absence in 75% sensitive isolates (1,619 genomes). We identified a total of 337 SNPs enriched across a pool of 207 genes from different chromosomes (Figure 3A). Normalized for the chromosome length, chromosome 13 harbored the maximum number of mutations followed by chromosome 14 (Figure 3B and Supplementary Table S3). This might be due to enhanced selection of patches of the chromosome 13 that favors the emergence of resistance. Among the genes that harbored the most mutations were a putative α-ß hydrolase (PF3D7_1328500) and a putative HECT domain–containing protein (PF3D7_0628100) (Supplementary Table S4). Of note were also an RNA-binding protein (PF3D7_0723900), a SET domain containing protein (PF3D7_0629700), autophagy-related protein 18 (PF3D7_1012900), and an AP2-domain transcription factor (PF3D7_1222400) (Supplementary Table S4). To further validate the co-existence of these distinct gene mutants with K13 mutations, we performed a chi-squared test to assess the relation between mutant/WT K13 with individual mutant/WT gene variants. We found that the association between K13 mutations and the candidate background gene mutations identified by us was statistically significant for all except the Pfmdr1 mutation (Supplementary Table S1). Although several mutations have already been reported to be selected in the background of K13 mutations like ferrodoxin (PF3D7_1318100) and ubiquitin C-terminal hydrolase (PF3D7_0722330), we report several novel mutations hitherto undescribed. An investigation of the possible pathways connected with these mutations using the GO analysis revealed SNPs enriched upon genes associated with endocytosis, host cell invasion/exit, and signal transduction (Figure 3C). These pathways have also been implicated as contributing to artemisinin resistance as per recent studies. Thus, it is possible that mutations in these genes (like those in K13) affect the protein functionality/abundance and may thus trigger/support resistance to artemisinin.

### Artemisinin Resistance Transcriptome: Investigation of Key Regulatory Factors and Processes

**FIGURE 4:**
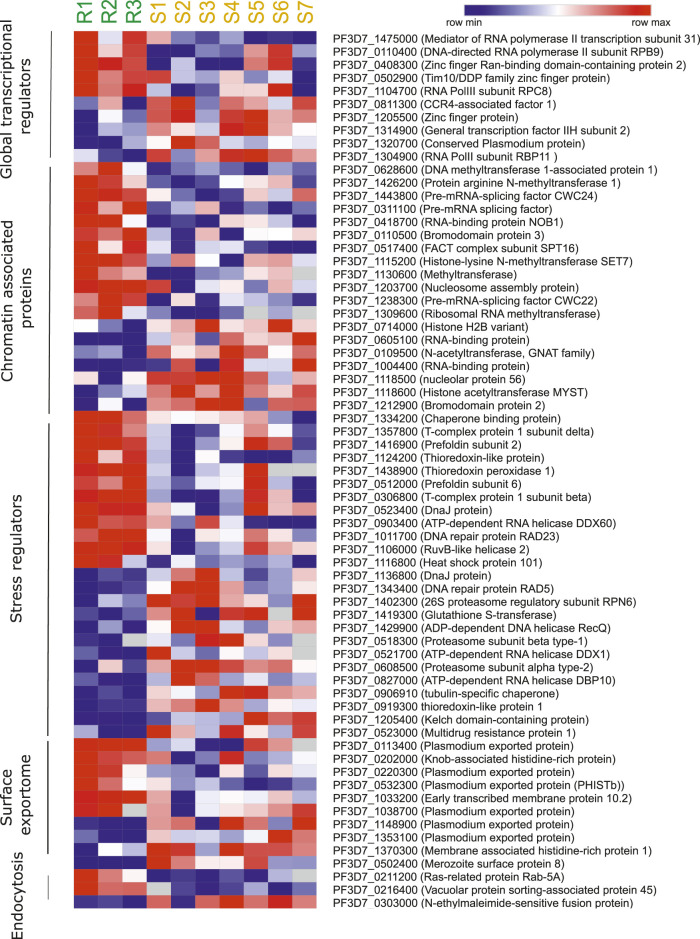
Artemisinin resistance transcriptome of different biological pathways. Heatmap representing the deregulated expression of select genes from key biological pathways in *Plasmodium* contributive to artemisinin resistance. The heatmap is generated from transcriptomic dataset of Mok et al., 2011 study comparing three artemisinin-resistant and seven artemisinin-sensitive parasite isolates. The genes were selected on the basis of their deregulation in this dataset and at least one other transcriptomic dataset that we reviewed (Mok et al., 2015 and Rocamora et al., 2018).

Next, we reviewed the transcriptome of artemisinin-sensitive and artemisinin-resistant parasites to identify the co-regulatory factors and processes. The datasets on field isolates from SEA in 2011 and 2015 (Mok et al.) were used as source for the *ex vivo* transcriptomes, whereas data from Rocamora et al. (2018) were used a source for the *in vitro–*selected artemisinin-resistant isolates (Mok et al., 2011; Mok et al., 2015; Rocamora et al., 2018). To investigate the transcriptional profile of genes from various pathways, we drafted a list of genes from each pathway from relevant publications and PlasmoDB gene MPMP database. Moreover, genes that showed at least two-fold differential expression and deregulated (both up and downregulated) across at least two of the three independent studies (Mok et al., 2011; Mok et al., 2015; Rocamora et al., 2018) were discussed and highlighted in the following sections. Heatmaps were generated for representing the deregulated expression of selected genes from key biological pathways in *Plasmodium* contributing to artemisinin resistance. Figure 4 is generated from transcriptomic dataset of the study of Mok et al. (2011) comparing three artemisinin-resistant and seven artemisinin-sensitive parasite isolates. The genes were selected on the basis of their deregulation in this dataset and at least one other transcriptomic dataset that we reviewed. Similarly, Supplementary Figure S2 is generated from the study of Mok et al. (2015). The genes were selected on the basis of their deregulation in the dataset of Mok et al. (2015) and at least one other transcriptomic dataset (Mok et al., 2015 or Rocomora et al., 2018), which we reviewed.

#### Global Transcriptional Regulators: Transcription Factors and Chromatin-Associated Factors

*P. falciparum* relies on a host of general [RNA polymerase II (RNA Pol II) core subunits/accessory proteins and general coactivators involved in initiation/elongation] and specific transcription-associated factors (apicomplexan AP2 factors, zinc finger proteins, and Helix turn helix motif proteins) for transcriptional control (Coulson et al., 2004; Balaji et al., 2005; Callebaut et al., 2005; Bischoff and Vaquero, 2010; Painter et al., 2011; Tuteja et al., 2011; Jeninga et al., 2019). Genetic ablation studies of a few of these factors, especially the ApiAP2 family of TFs, have hinted at strong roles of proteins in invasion into host red blood cells, governing the expression of exported proteins, gametocytogenesis, and liver-stage development (Iwanaga et al., 2012; Modrzynska et al., 2017; Santos et al., 2017; Van Biljon et al., 2019). Given the wide range of processes that transcription factors can govern, we were interested in identifying changes in their expression profile associated with artemisinin resistance. We procured a list of transcription factors, transcription-associated proteins, and chromatin-associated factors that aid in transcription from the PlasmoDB gene search database (Aurrecoechea et al., 2009). A list of high confidence transcription factors and associated proteins was also referenced from a bioinformatic study by Vaquero et al*.* (Bischoff and Vaquero, 2010). Our cross-dataset comparison revealed numerous factors that were commonly found to be deregulated across multiple studies. The RNA Pol II subunit RBP9 (PF3D7_0110400) and zinc finger protein (Ran-binding domain containing) (PF3D7_0408300) were upregulated, and CCR4-associated factor 1 (PF3D7_0811300), zinc finger protein (PF3D7_1205500), RNA Pol II subunit RBP11 (PF3D7_1304900), and conserved protein (PF3D7_1320700) were found to be downregulated in artemisinin-resistant parasites (Figure 4). In general, we see a strong correlation of the deregulation of multiple ZnF proteins and DNA-dependent RNA Pol II subunits with the artemisinin resistance phenotype both in the field and *in vitro* settings (Supplementary Table S5). These factors are higher up in the gene expression/regulation hierarchy and may have numerous genes (and consequently biological processes) under their direct regulation. Thus, they would be of value to investigate and understand the widespread transcriptional deregulation that precedes the establishment of artemisinin resistance in parasites.

We surveyed literature related to chromatin-associated proteins (CAPs) in *P. falciparum*. Primary gene set was procured from the dataset by Batugedara et al. (Batugedara et al., 2020), who utilized a combination of *in silico* methods and Chromatin Enrichment for Proteomics (ChEP) to identify a bona fide set of CAPs (Batugedara et al., 2020). These CAPS were identified to enrich for various biological processes in GO analysis, *viz*., cell division, mRNA processing, protein modifications, ubiquitin-associated, and chromatin-associated (Batugedara et al., 2020). We decided to filter for CAPs that were enriched for chromatin specific functions exclusively, considering their potential role in regulation of gene expression. We also supplemented our dataset of candidate genes from the MPMP database, filtering specifically for chromatin modifying proteins (Aurrecoechea et al., 2009). We finally curated a set of 57 proteins belonging to various classes of epigenetic modifiers (histone acetyltransferase, histone deacetylase, histone lysine methyltransferase, protein arginine methyltransferase, and histone demethylase), chromatin remodelers, and nuclear architecture proteins. The CAPs were observed to be often downregulated in association with artemisinin-resistant parasites. Importantly, the RNA-binding protein NOB1 (PF3D7_0418700), a putative methyltransferase (PF3D7_1130600), a pre-mRNA splicing factor CWC22 (PF3D7_1238300), and a ribosomal RNA methyl transferase (PF3D7_1309600) were all found to be upregulated (Figure 4 and Table 2). Interestingly, the histone acetyltransferase MYST (PF3D7_1118600) and the GNAT family member acetyltransferase (PF3D7_0109500) were downregulated in the resistant parasites (Supplementary Table S5). The histone H2B variant (PF3D7_0714000), two putative RNA-binding proteins (PF3D7_0605100 and PF3D7_1004400), and a putative bromodomain protein (PF3D7_1212900) were also downregulated in artemisinin-resistant parasites (Supplementary Table S5). The deregulation of histone PTM code modifiers allows for widespread changes to take place in the gene expression profile, which may hold true for resistant parasites as well.

**TABLE 2:** Pathways and gene implicated in artemisinin resistance. Genes in red are upregulated in resistant parasites, whereas those represented in blue are downregulated.

Processes/Pathways	Key genes deregulated
Transcription Factors	DNA-directed RNA polymerase II subunit RPB9 (PF3D7_0110400)
	Zinc finger Ran-binding domain–containing protein 2 (PF3D7_0408300)
	Tim10/DDP family zinc finger protein (PF3D7_0502900)
	PF3D7_0811300 (CCR4-associated factor 1)
Chromatin-Associated Factors	Methyltransferase (PF3D7_1130600, PF3D7_1115200, PF3D7_1309600, PF3D7_1426200)
	Putative DNMT1 associated protein (PF3D7_0628600)
	Pre-mRNA splicing factor (PF3D7_1238300, PF3D7_1443800, PF3D7_0311100)
	Bromodomain protein 3 (PF3D7_0110500)
	Nucleosome assembly protein (PF3D7_1203700).
	Histone acetyl transferase MYST (PF3D7_1118600)
	GNAT family member acetyl transferase (PF3D7_0109500
	RNA-binding proteins (PF3D7_0605100 and PF3D7_1004400)
	Bromodomain protein (PF3D7_1212900)
Global stress regulators	DNA repair protein RAD23 (PF3D7_1011700)
	RAD54 DNA recombination and repair protein (PF3D7_1343400)
	The T-complex 1 subunit beta (PF3D7_0306800) and subunit delta (PF3D7_1357800)
	Prefoldin subunit 6 (PF3D7_0512000)
	Prefoldin subunit 2 (PF3D7_1416900)
	DnaJ protein (PF3D7_0523400)
	Thioredoxin peroxidase 1 (PF3D7_1438900) Thioredoxin-like protein (PF3D7_1124200)
Surface exportome	PHISTb (PF3D7_0532300, PF3D7_0731300, PF3D7_1477500 trophozoite exported protein 1 (PF3D7_0603400)
Endocytosis	Ras-related protein Rab-5a (PF3D7_0211200)
	Vacuolar protein sorting–associated protein 45 (PF3D7_0216400)
	Multidrug resistance protein 1 (MDR1) (PF3D7_0523000)
	Kelch domain–containing protein (PF3D7_1205400)
Metabolic enzymes	Glycine cleavage H protein (PF3D7_1132900)
	Glycine cleavage T protein (PF3D7_1365500)

#### Stress Regulators

The extreme reactive nature of artemisinin leads to the build of reactive oxygen species and toxic misfolded proteins in the parasite cell (Mok et al., 2015; Rocamora et al., 2018; Suresh and Haldar, 2018). In the eventuality of insufficient management or clearance of these toxic aggregates, the parasite succumbs. Furthermore, artemisinin is also speculated to impart direct damage to the parasite DNA either by free radical mechanisms or by forming direct adducts (Gopalakrishnan and Kumar, 2015; Kadioglu et al., 2017). Thus, the damage to proteins and DNA are the major consequences of artemisinin exposure in parasites and need to be dealt with rapidly. As a consequence, the parasite must invest significantly in mechanisms that can help it survive under inhospitable conditions and proliferate on the host cell resources.

RNA helicases in *P. falciparum* have been reported to participate in major RNA metabolic processes including ribosome biogenesis, transcriptional control and fidelity, splicing, and translation (Tuteja and Pradhan, 2006; Tuteja, 2010). These are the biological processes that see major dysregulation during the artemisinin treatment. Studies suggest the deregulation of RNA helicases upon exposure to stresses, especially therapeutic (chloroquine) (Thélu et al., 1994). Thus, it becomes especially relevant to study their expression profiles in relevance to artemisinin resistance. The ATP-dependent RNA Helicase DDX60 (PF3D7_0903400) and DNA repair protein RAD23 (PF3D7_1011700) and RuvB like helicase (PF3D7_1106000) were found to be upregulated in the artemisinin-resistant parasites from the study of Mok et al. (2011) and in the *in vitro* generated resistant line 6A-R (Rocamora, 2018) (Supplementary Table S5). Conversely, the ATP-dependent RNA helicase DBP9 (PF3D7_1429900), the DEAD box helicase PF3D7_1439100, and the RAD54 DNA recombination and repair protein (PF3D7_1343400) were downregulated in the resistant parasites (Supplementary Table S5). Because helicases tend to perform a diversity of regulatory functions, it would be important to investigate them individually for any specific contribution to artemisinin resistance.

Heat-shock proteins (HSPs)/chaperones are among the prime candidates for cytoprotective functions and cellular repairs (Daniyan et al., 2019). HSPs play a crucial role as biomolecular chaperones by performing various functions like folding, unfolding, assembly of proteins, and transport of proteins into correct subcellular compartments (Joshi et al., 1992; Akide-Ndunge et al., 2009). Artemisinin exposure also damages the parasite proteome extensively and compromises cellular functions (Prieto et al., 2008). This makes the functions of homeostatic chaperone protein even more important in parasite survival. The chaperone proteins and the protein homeostasis-associated machinery are reported to be extensively involved in emergence of artemisinin resistance and thus interesting molecular candidates to follow (Rawat et al., 2021). The T-complex 1 subunit beta (PF3D7_0306800) and subunit delta (PF3D7_1357800), putative chaperone binding protein (PF3D7_1334200), prefoldin subunit 6 (PF3D7_0512000), and DnaJ protein (PF3D7_0523400), Hsp101 (PF3D7_1116800; ClpB2), and prefoldin subunit 2 (PF3D7_1416900) were upregulated in artemisinin-resistant parasites (Supplementary Table S5). Interestingly, a tubulin specific chaperone (PF3D7_0906910) and DnaJ protein (PF3D7_1136800) were downregulated in resistant isolates (Supplementary Table S5). The upregulation of numerous proteostasis factors (especially chaperones) has been highlighted for their role in mitigating the damage invoked by artemisinin and proteostasis.

Redox systems are known to play an important role in the survival of parasites under any oxidative stress conditions (Kehr et al., 2010; Nepveu and Turrini, 2013). *P. falciparum* possesses thioredoxin and glutathione redox systems, which constitutes the thiol-based antioxidant defense system along with superoxide dismutase (Jortzik and Becker, 2012). Recent studies have highlighted the significance of these pathways in artemisinin-resistant parasites because they play an important role in the maintenance of homeostasis in presence of free radicals during artemisinin activation (Rocamora et al., 2018). Rocomora et al. (2018) identified several genes involved in redox metabolism to be upregulated in *in vitro* lab-generated resistant strain like thioredoxin-like protein (PF3D7_1124200), thioredoxin 1 (PF3D7_1457200), thioredoxin peroxidase 1 (PF3D7_1438900), and glutathione reductase (PF3D7_1419800) (Supplementary Table S5). Surprisingly, most of the members of proteins belonging to thioredoxin and glutathione systems were found to be downregulated in the clinical isolates of resistant parasites, except thioredoxin peroxidase 1 (PF3D7_1438900) and thioredoxin-like protein (PF3D7_1124200) (Supplementary Table S5). Understanding the role of redox proteins in oxidative stress will be useful in targeting these proteins to overcome artemisinin resistance.

#### Surface Exportome

The parasite employs some remarkable strategies of host cell remodeling to enable it to thrive and survive in this niche environment (Schulze et al., 2015; de Koning-Ward et al., 2016; Warncke et al., 2019). It has been shown that ∼8%–10% of the *P. falciparum* proteome is exported; collectively, these proteins are referred to as the “exportome” (Mundwiler-Pachlatko and Beck, 2013). The parasite-derived exported proteins play an important role in determining the rigidity of the RBC, permeating it for nutrients and metabolites along with imparting new attributes such as adherence, and clumping to avoid immune and splenic clearance (Mundwiler-Pachlatko and Beck, 2013). Antigenic gene families such as PfEMP1, rifins, stevors, surfins, and PfMC-2TM are known to cause the phenomenon such as cytoadherence, rosetting, and clumping (Dzikowski and Deitsch, 2009). Whereas, *Plasmodium* helical interspersed subtelomeric (PHIST) family proteins are known to remodel the RBC surface through incorporation into host cytoskeleton (Prajapati and Singh, 2013; Warncke et al., 2016). It is known that artemisinin-resistant parasites having K13 mutation with elevated PI3P show enriched PfEMP1 containing export proteome (Mbengue et al., 2015). This provides resistant parasites with better cytoadherence and hence successful immune evasion as compared to sensitive ones (Bhattacharjee et al., 2018). In concordance, we observed upregulation of a large number of exported proteins PF3D7_1038700 and PF3D7_0220300 (exported proteins of unknown function), a knob-associated histidine-rich protein (PF3D7_0202000), the early transcribed membrane protein 10.2 (PF3D7_1033200), and several HISTb proteins (Supplementary Table S5). On the other hand, the merozoite surface protein 8 (PF3D7_0502400), two exported proteins of unknown function (PF3D7_1148900 and PF3D7_1353100), and the membrane-associated histidine-rich protein 1 (PF3D7_1370300) were found to be downregulated in artemisinin-resistant parasites (Supplementary Table S5). Although we did observe dramatic changes in expression of exported proteins associated with artemisinin resistance especially in the *in vitro*–selected resistant lines from the study of Rocamora et al. (2018), these changes were not found to be conserved across observations made from other studies.

#### Cytostomal Invagination Pathway

*Plasmodium* utilizes cytostome to imports chunks of host cell cytoplasm rich in hemoglobin *via* endocytic process which subsequently fuses to the food vacuole for further processing (Spielmann et al., 2020). Recent studies have highlighted the localisation of K13 protein in close proximity of the vesicular complex (Birnbaum et al., 2020). It was shown that parasites with inactivated K13 or a resistance-conferring K13 mutation displayed reduced hemoglobin endocytosis and resistance to artemisinin (Birnbaum et al., 2020). ARTs are activated by degradation products of hemoglobin. Hence, reduced activity of K13 and its interactors diminishes hemoglobin endocytosis and thereby artemisinin activation, resulting in parasite resistance (Siddiqui et al., 2017). This suggests that the process of endocytosis is critical to resistance generation in *P. falciparum*. In this regard, we were interested to investigate the role of endocytosis-related proteins across the artemisinin-resistant *Plasmodium* parasites. Our analysis found the Ras-related protein Rab-5a (PF3D7_0211200) and the vacuolar protein sorting–associated protein 45 (PF3D7_0216400) upregulated and a solitary N-ethylmaleimide-sensitive fusion protein (PF3D7_0303000) downregulated in the artemisinin-resistant parasites (Supplementary Table S5). Interestingly, not many endosome-associated proteins were found to be transcriptionally deregulated in artemisinin-resistant parasites. Fewer still were deregulated across multiple datasets.

#### Metabolic Pathways

**FIGURE 5:**
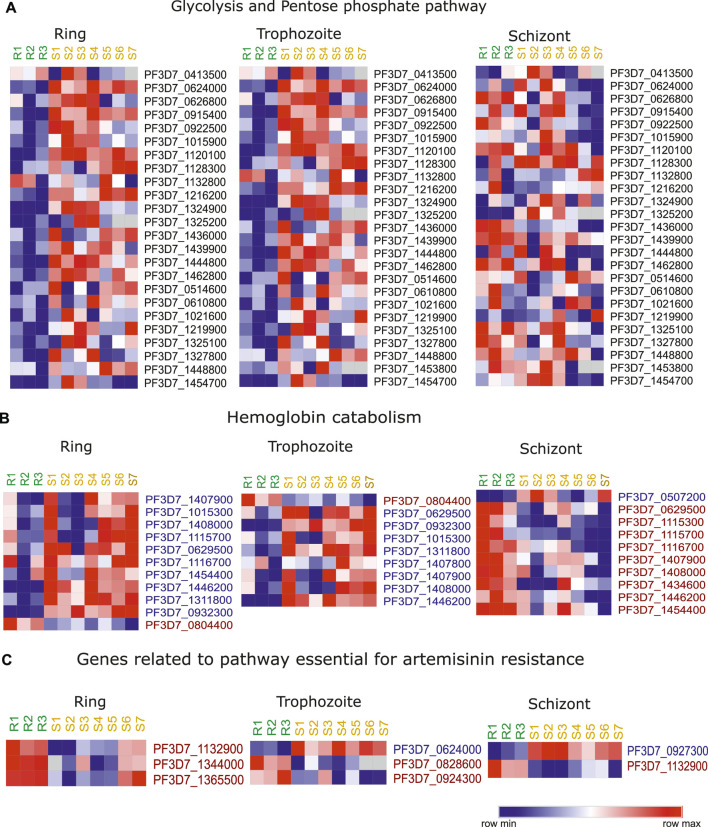
Artemisinin resistance transcriptome for hemoglobin catabolism related genes and metabolic genes. Heatmap representing the stage specific trends in deregulation of expression of key genes implicated in **(A)** glycolysis and pentose phosphate pathway, **(B)** hemoglobin catabolism, and **(C)** genes related to pathway important for artemisinin resistance. The heatmap is generated from the expression values from the study of Mok et al. (2011). The list of genes themselves has been selected on the basis of deregulation of these genes across artemisinin-resistant vs. artemisinin-sensitive isolates in this and at least one other transcriptomic dataset reviewed in this study. R1–R3 represent the three artemisinin-resistant isolates, whereas S1–S7 represent the artemisinin-sensitive isolates.

*Plasmodium* is known to undergo metabolic alterations under different environmental and physiological conditions that are regulated by genetic and epigenetic mechanism (Lang-Unnasch and Murphy, 1998; Srivastava et al., 2016; Tewari et al., 2020). Being extensively adapted for a parasitic mode of life *Plasmodium* relies on the host for nutrients (Olszewski et al., 2009; Kafsack and Llinás, 2010; Zuzarte-Luís and Mota, 2018; Tougan et al., 2020). Several reports have indicated the role of metabolism in *P. falciparum* artemisinin resistance emergence (Carey et al., 2017; Guggisberg et al., 2018; Mok et al., 2021). Upon artemisinin treatment, *P. falciparum* is known to enter morphologically distinct quiescent stage that is characterized by reduced metabolism as a mean to resist unfavorable conditions (Teuscher et al., 2010; Chen et al., 2014). Metabolomics studies have identified the accumulation of glutathione and its precursor, gamma-glutamylcysteine, and significant depletion of one other putative metabolite in resistant strains (Siddiqui et al., 2017). Interestingly, dihydroartemisinin (DHA) treatment interferes with hemoglobin catabolism and pyridine biosynthesis (Cobbold et al., 2016). Covelli et al. in in their metabolomics study found significant reduction in the levels of Orate, which is a metabolite product of pyrimidine biosynthesis (Covelli et al., 2016). In addition, metabolites derived from lipid and cholesterol were significantly higher in DHA-exposed sensitive parasites (Covelli et al., 2016). Under DHA-induced dormancy, most of the metabolic pathways were found to be downregulated except fatty acid and pyruvate metabolic pathways that were active during phenomenon of dormancy (Chen et al., 2014). Inhibition studies of fatty acid and pyruvate metabolic pathway have been corelated with delayed recovery of dormant parasites (Chen et al., 2014). Hence, despite several studies available in the literature, the mechanism by which these metabolites conferring resistance to parasites is unclear. We looked at the transcriptional profile of the metabolic pathway genes in artemisinin-resistant isolates. We observed a clear downregulation of major metabolic pathways (glycolysis and pentose phosphate pathway) during intra erythrocytic life cycle in artemisinin-resistant parasites (Figure 5A).

Hemoglobin (Hb) digestion within the food vacuole results in the supply of amino acid to the parasite (Goldberg et al., 1990). Recent studies have shown that reduced hemoglobin digestion is responsible for reduced sensitivity to artemisinin, owing to lower availability of Fe^2+^ ions (generated by Hb catabolism) (Yang et al., 2019). Therefore, we looked at the stage specific expression of the genes that are involved in the digestion of hemoglobin in the dataset of Mok et al. (2011). Interestingly, these genes were downregulated in the resistant parasites during ring and trophozoite stages and upregulated during the schizont stage (Figure 5B). The downregulation of genes like aminopeptidase P (PF3D7_1454400), M1-family alanyl aminopeptidase (PF3D7_1311800), and cysteine proteinase falcipain 2a (PF3D7_1115700) is indicative of the fact that resistant parasites have reduced hemoglobin digestion (Figure 5B). Importantly, knockout of cysteine protease falcipain 2a (PF3D7_1115700) is reported to result in delayed sensitivity of parasites to artemisinin (Siddiqui et al., 2018). Moreover, mutations in cysteine falcipain 2a gene are also identified in *in vitro*–selected artemisinin-resistant parasites (Siddiqui et al., 2018). We looked at the expression level of these genes coding the enzyme vital for the resistant parasites and compared it to the sensitive parasites (Carey et al., 2017). Interestingly, out of the 11 genes reported to be essential for the artemisinin resistance, three genes were found to be highly expressed in resistant parasites: glycine cleavage H protein (PF3D7_1132900), glycine cleavage T protein (PF3D7_1365500), and aminomethyltransferase (PF3D7_1344000) during the ring stage of the parasites (Figure 5C). Similarly, we found folate transporter 1 (PF3D7_0828600) and thiamine pyrophosphokinase (PF3D7_0924300) to be upregulated and hexokinase (PF3D7_0624000) to be downregulated in resistant isolates from the study of Mok et al. (2011) during trophozoite stage (Figure 5C). Among chemical reactions unique to sensitive parasites, pyruvate kinase (PF3D7_0626800) was found to be significantly downregulated in resistant parasites. Two lipid metabolism-related genes phosphatidylinositol transfer protein (PF3D7_1351000) and phosphoinositide-binding protein (PF3D7_0720700) were found to be upregulated in artemisinin-resistant parasites.

### Artemisinin Resistance Proteome

Similar to the transcriptome, the proteome of *P. falciparum* varies with the stage of the parasite, and this has been the subject of many proteomic studies. Because proteins are the actual effector molecules in the cells, in recent years, the focus has shifted to a careful proteomic analysis of the artemisinin-resistant parasites. A finely tuned protein turnover machinery can help parasite to adapt under changing environment and drug treatment and should thus be properly studied to identify factors that can be crucial and be targeted. Artemisinin resistance mediated by K13 mutations is known to result in a dramatic upregulation of PI3P molecules and its vesicular structures (Mbengue et al., 2015; Bhattacharjee et al., 2018). Global peptidomics analysis suggested the lower levels of peptides derived from hemoglobin (HBα and HBβ) in artemisinin-resistant parasites (Siddiqui et al., 2017). Interestingly, hemoglobin catabolism–related genes were found to be downregulated in artemisinin-resistant lines that show a protein level validation of the downregulation in hemoglobin catabolism (Siddiqui et al., 2017). Recently, Ismail et al. (2016) identified protein targets of artemisinin drug using click chemistry (Ismail et al., 2016b). Interestingly, some of the proteins like M1-family alanyl aminopeptidase (PF3D7_1311800), Plasmepsin I (PF3D7_1407900), and II (PF3D7_1408000), which assist in hemoglobin digestion, were found to be direct target of artemisinin (Ismail et al., 2016b). This establishes a potential link between downregulation in hemoglobin peptide on DHA treatment and direct alkylation and inhibition of these proteases by artemisinin drug.

Bhattacharjee et al. recently reported the presence of the amplified PI3P vesicles, which helps in neutralizing the protein damage due to artemisinin (Bhattacharjee et al., 2018). These vesicles house proteins like K13, PfEMP1, and BiP and other proteins useful for maintaining homeostasis during artemisinin treatment. Out of the 502 proteins identified in their proteomic analysis of PI3P vesicles, approximately 72 are also reported to be upregulated at transcript level in transcriptomic study of artemisinin-resistant patient samples (Birnbaum et al., 2020).

## Conclusion

A close inspection of the genomic and transcriptomic features of artemisinin-resistant isolates has identified/reaffirmed peculiar trends of genetic selection and gene expression patterns. K13 mutations still predominantly mark artemisinin resistance phenotype with the C580Y, R539T, and Y493H genotype in decreasing order of global prevalence (Ménard et al., 2016). Although the K13 mutations are dominant in the Southeast Asian countries, there is minor presence in Africa as well. African nations, however, report a distinct K13 mutations A578S being the major player (Ménard et al., 2016). Interestingly, only a few isolates showed double/triple K13 mutations perhaps, owing to the strong destabilizing effect individual mutations impose on parasite viability. In our efforts to explore the genetic markers/polymorphisms supporting K13 mutations, we identify a strong selection against a backdrop of Pfcrt mutations (K76T, A220S, I536T, and R571I) but not so much with Pfmdr mutations (only Y184F seemed to co-exist with K13 mutations). Pf mutations seem to have reduced in prevalence over decades of discontinued usage of chloroquine in the region. It might be possible that the lack of common Pfcrt markers over the recent years has reduced the selection of common K13 mutations, thus explaining their poor presence in the region while also allowing for novel mutations in K13 to emerge. Finally, we interrogated the co-existence of K13 mutations with other key SNPs in the genomic background of resistant isolates. With a 75% resistant vs. 25% sensitive filter, we identified a host of SNPs that exist alongside the three prominent K13 mutations. An α-ß hydrolase and a putative HECT domain–containing gene were found to harbor the maximum number of SNPs, with chromosome 13 bearing the maximum SNP load. It is quite possible that these genes showing high mutational burden may be playing some role as background mutations to support the Kelch-resistant mutation. Significant enrichment of endocytosis and host cell entry/egress pathways was noted. The spatial and temporal selection dynamics of genetic polymorphisms and their interplay toward deciding the fate of drug therapy across the globe shall be very interesting to follow up further.

A thorough investigation of the transcriptomic datasets of the artemisinin-resistant field isolates and *in vitro*–selected strains has helped us narrow down on key genes whose expression changes may be interesting to follow up for validation and subsequent studies. In our top-down approach, we focused on transcription regulatory protein, the broader chromatin associated proteome and stress response factors particularly because of their speculated roles in bringing about artemisinin resistance in the parasite. We observed a consistent deregulation of several transcription factors (ZnF proteins) and numerous epigenetic regulator (PfMYST and PfSET7) proteins that dictate the temporal expression of genes. Owing to their higher placement in the hierarchy of gene expression and a plethora of biological pathways under their regulation, some of these may be key to the broader deregulation that is characteristic of artemisinin resistance. We also found a strong deregulation of RNA Pol II–associated proteins across the IDC that may account for the redistribution of RNA expression observed in resistance (low in early stages and a burst of transcriptional activity toward the mature stages). We further looked out for stress responsive factors in the parasites. We observed a robust change in the expression profile of specific ATP-dependent RNA helicases that govern key processes like transcriptional fidelity and splicing. This may, in turn, have implications on the overall RNA output and quality control. HSPs were found to be significantly among the upregulated genes cohort testimony of their role in controlling protein damage in resistant isolates. An interesting aspect that we wanted to investigate was the expression of exported protein genes, but, although these genes show deregulation in individual datasets, we did not observe a very consistent change across studies. Expression of exported proteins is often very sensitive to changes in environmental parameters that change across studies dramatically.

Our investigation of the metabolic pathway genes expression showed consistent downregulation of glycolytic and pentose phosphate pathway–associated genes. This strongly reiterates the metabolic slowdown that is characteristically associated with the ring stage parasites that are resistant to artemisinin. Also notable is the consistent downregulation of numerous aminopeptidases associated with hemoglobin catabolism. This follows course with numerous recent investigations, highlighting reduction of heme metabolism to be associated with artemisinin resistance in parasites. In addition, of note was the dramatic upregulation of genes associated with the folate metabolism in resistant parasites. We also reaffirm the overexpression of numerous redox pathway–associated genes in multiple studies. Our analysis highlights the aberrant expression of specific genes from pathways that have been implicated in mediating resistance. Numerous such examples have been highlighted in Table 2.

There is still a dearth of proteomic data relevant to investigating artemisinin resistance. However, the handful of studies that exist seem to suggest the parasite’s attempts to reduce hemoglobin catabolism perhaps in attempts to suppress activation of drug. This also matches with transcriptomic investigations that seem to hint at a decline in the abundance of hemoglobin catabolism enzymes. Simultaneously, the parasite does seem to enrich its proteome (especially in PI3P marked vesicles) with proteostatic factors associated with chaperoning, translation, and quality control functions. More proteomic studies need follow to better understand the interplay between well documented transcriptional changes and the little known proteome.

## Data Availability

The original contributions presented in the study are included in the article/Supplementary Material, further inquiries can be directed to the corresponding authors.

## References

[B1] Akide-Ndunge O. B. Tambini E. Giribaldi G. McMillan P. J. Müller S. Arese P. (2009). Co-ordinated Stage-dependent Enhancement of Plasmodium Falciparum Antioxidant Enzymes and Heat Shock Protein Expression in Parasites Growing in Oxidatively Stressed or G6PD-Deficient Red Blood Cells. Malar. J. 8, 113–115. 10.1186/1475-2875-8-113 19480682 PMC2696464

[B2] Antony H. A. Das S. Parija S. C. Padhi S. (2016). Sequence Analysis of Pfcrt and Pfmdr1 Genes and its Association with Chloroquine Resistance in Southeast Indian Plasmodium Falciparum Isolates. Genomics Data 8, 85–90. 10.1016/j.gdata.2016.04.010 27222806 PMC4856815

[B3] Ariey F. Witkowski B. Amaratunga C. Beghain J. Langlois A.-C. Khim N. (2014). A Molecular Marker of Artemisinin-Resistant Plasmodium Falciparum Malaria. Nature 505, 50–55. 10.1038/nature12876 24352242 PMC5007947

[B4] Aurrecoechea C. Brestelli J. Brunk B. P. Dommer J. Fischer S. Gajria B. (2009). PlasmoDB: a Functional Genomic Database for Malaria Parasites. Nucleic Acids Res. 37, D539–D543. 10.1093/nar/gkn814 18957442 PMC2686598

[B5] Bakhiet A. M. A. Abdelraheem M. H. Kheir A. Omer S. Gismelseed L. Abdel-Muhsin A.-M. A. (2019). Evolution of Plasmodium Falciparum Drug Resistance Genes Following Artemisinin Combination Therapy in Sudan. Trans. R. Soc. Trop. Med. Hyg. 113, 693–700. 10.1093/trstmh/trz059 31369106

[B6] Balaji S. Babu M. M. Iyer L. M. Aravind L. J. N. a. r. (2005). Discovery of the Principal Specific Transcription Factors of Apicomplexa and Their Implication for the Evolution of the AP2-Integrase DNA Binding Domains. Nucleic Acids Res. 33, 3994–4006. 10.1093/nar/gki709 16040597 PMC1178005

[B7] Batugedara G. Lu X. M. Saraf A. Sardiu M. E. Cort A. Abel S. (2020). The Chromatin Bound Proteome of the Human Malaria Parasite. Microb. Genom 6, e000327. 10.1099/mgen.0.000327 PMC7067212 32017676

[B8] Bhattacharjee S. Coppens I. Mbengue A. Suresh N. Ghorbal M. Slouka Z. (2018). Remodeling of the Malaria Parasite and Host Human Red Cell by Vesicle Amplification that Induces Artemisinin Resistance. Blood 131, 1234–1247. 10.1182/blood-2017-11-814665 29363540 PMC5855022

[B9] Birnbaum J. Scharf S. Schmidt S. Jonscher E. Hoeijmakers W. A. M. Flemming S. (2020). A Kelch13-Defined Endocytosis Pathway Mediates Artemisinin Resistance in Malaria Parasites. Science 367, 51–59. 10.1126/science.aax4735 31896710

[B10] Bischoff E. Vaquero C. (2010). In Silico and Biological Survey of Transcription-Associated Proteins Implicated in the Transcriptional Machinery during the Erythrocytic Development of Plasmodium Falciparum. BMC Genomics 11, 34–20. 10.1186/1471-2164-11-34 20078850 PMC2821373

[B11] Callebaut I. Prat K. Meurice E. Mornon J. P. Tomavo S. (2005). Prediction of the General Transcription Factors Associated with RNA Polymerase II in Plasmodium Falciparum: Conserved Features and Differences Relative to Other Eukaryotes. BMC Genomics 6, 100–120. 10.1186/1471-2164-6-100 16042788 PMC1199594

[B12] Carey M. A. Papin J. A. Guler J. L. (2017). Novel Plasmodium Falciparum Metabolic Network Reconstruction Identifies Shifts Associated with Clinical Antimalarial Resistance. BMC Genomics 18, 543–619. 10.1186/s12864-017-3905-1 28724354 PMC5518114

[B13] Chan Y. Walmsley R. P. (1997). Learning and Understanding the Kruskal-Wallis One-Way Analysis-Of-Variance-By-Ranks Test for Differences Among Three or More Independent Groups. Phys. Ther. 77, 1755–1761. 10.1093/ptj/77.12.1755 9413454

[B14] Chen N. LaCrue A. N. Teuscher F. Waters N. C. Gatton M. L. Kyle D. E. (2014). Fatty Acid Synthesis and Pyruvate Metabolism Pathways Remain Active in Dihydroartemisinin-Induced Dormant Ring Stages of Plasmodium Falciparum. Antimicrob. Agents Chemother. 58, 4773–4781. 10.1128/aac.02647-14 24913167 PMC4135995

[B15] Cingolani P. Platts A. Wang L. L. Coon M. Nguyen T. Wang L. (2012). A Program for Annotating and Predicting the Effects of Single Nucleotide Polymorphisms, SnpEff. Fly 6, 80–92. 10.4161/fly.19695 22728672 PMC3679285

[B16] Cobbold S. A. Chua H. H. Nijagal B. Creek D. J. Ralph S. A. McConville M. J. (2016). Metabolic Dysregulation Induced inPlasmodium Falciparumby Dihydroartemisinin and Other Front-Line Antimalarial Drugs. J. Infect. Dis. 213, 276–286. 10.1093/infdis/jiv372 26150544

[B17] Coppée R. Jeffares D. C. Miteva M. A. Sabbagh A. Clain J. (2019). Comparative Structural and Evolutionary Analyses Predict Functional Sites in the Artemisinin Resistance Malaria Protein K13. Sci. Rep. 9, 10675–10717. 10.1038/s41598-019-47034-6 31337835 PMC6650413

[B18] Coulson R. M. R. Hall N. Ouzounis C. A. (2004). Comparative Genomics of Transcriptional Control in the Human Malaria Parasite Plasmodium Falciparum. Genome Res. 14, 1548–1554. 10.1101/gr.2218604 15256513 PMC509263

[B19] Covelli V. Cooper J. Carey M. Guler J. (2016). Metabolomics for the *In Vitro* Study of Artemisinin-Resistant Malaria Parasites. Paper presented Open Forum Infect. Dis. 3.

[B20] Daniyan M. O. Przyborski J. M. Shonhai A. (2019). Partners in Mischief: Functional Networks of Heat Shock Proteins of Plasmodium Falciparum and Their Influence on Parasite Virulence. Biomolecules 9, 295. 10.3390/biom9070295 PMC6681276 31340488

[B21] de Koning-Ward T. F. Dixon M. W. A. Tilley L. Gilson P. R. (2016). Plasmodium Species: Master Renovators of Their Host Cells. Nat. Rev. Microbiol. 14, 494–507. 10.1038/nrmicro.2016.79 27374802

[B22] Dondorp A. M. Nosten F. Yi P. Das D. Phyo A. P. Tarning J. (2009). Artemisinin Resistance inPlasmodium falciparumMalaria. N. Engl. J. Med. 361, 455–467. 10.1056/nejmoa0808859 19641202 PMC3495232

[B23] Dwivedi A. Reynes C. Kuehn A. Roche D. B. Khim N. Hebrard M. (2017). Functional Analysis of Plasmodium Falciparum Subpopulations Associated with Artemisinin Resistance in Cambodia. Malar. J. 16, 493–517. 10.1186/s12936-017-2140-1 29258508 PMC5735551

[B24] Dzikowski R. Deitsch K. W. (2009). Genetics of Antigenic Variation in Plasmodium Falciparum. Curr. Genet. 55, 103–110. 10.1007/s00294-009-0233-2 19242694 PMC3640992

[B25] Goldberg D. E. Slater A. F. Cerami A. Henderson G. B. (1990). Hemoglobin Degradation in the Malaria Parasite Plasmodium Falciparum: an Ordered Process in a Unique Organelle. Proc. Natl. Acad. Sci. 87, 2931–2935. 10.1073/pnas.87.8.2931 2183218 PMC53807

[B26] Gopalakrishnan A. M. Kumar N. (2015). Antimalarial Action of Artesunate Involves DNA Damage Mediated by Reactive Oxygen Species. Antimicrob. Agents Chemother. 59, 317–325. 10.1128/aac.03663-14 25348537 PMC4291367

[B27] Guggisberg A. M. Frasse P. M. Jezewski A. J. Kafai N. M. Gandhi A. Y. Erlinger S. J. (2018). Suppression of Drug Resistance Reveals a Genetic Mechanism of Metabolic Plasticity in Malaria Parasites. MBio 9, e01193. 10.1128/mbio.01193-18 30425143 PMC6234871

[B28] Ismail H. M. Barton V. E. Panchana M. Charoensutthivarakul S. Biagini G. A. Ward S. A. (2016). A Click Chemistry-Based Proteomic Approach Reveals that 1,2,4-Trioxolane and Artemisinin Antimalarials Share a Common Protein Alkylation Profile. Angew. Chem. Int. Ed. 55, 6401–6405. 10.1002/anie.201512062 PMC4934138 27089538

[B29] Ismail H. M. Barton V. Phanchana M. Charoensutthivarakul S. Wong M. H. L. Hemingway J. (2016). Artemisinin Activity-Based Probes Identify Multiple Molecular Targets within the Asexual Stage of the Malaria Parasites Plasmodium Falciparum 3D7. Proc. Natl. Acad. Sci. USA 113, 2080–2085. 10.1073/pnas.1600459113 26858419 PMC4776496

[B30] Iwanaga S. Kaneko I. Kato T. Yuda M. (2012). Identification of an AP2-Family Protein that Is Critical for Malaria Liver Stage Development. PLoS ONE 7, e47557. 10.1371/journal.pone.0047557 23144823 PMC3492389

[B31] Jeninga M. Quinn J. Petter M. (2019). ApiAP2 Transcription Factors in Apicomplexan Parasites. Pathogens 8, 47. 10.3390/pathogens8020047 PMC6631176 30959972

[B32] Jiang T. Huang Y. Cheng W. Sun Y. Wei W. Wu K. (2021). Multiple Single-Nucleotide Polymorphism Detection for Antimalarial Pyrimethamine Resistance via Allele-specific PCR Coupled with Gold Nanoparticle-Based Lateral Flow Biosensor. Antimicrob. Agents Chemother. 65, e01063–20. 10.1128/aac.01063-20 33361302 PMC8092547

[B33] Jortzik E. Becker K. (2012). Thioredoxin and Glutathione Systems in Plasmodium Falciparum. Int. J. Med. Microbiol. 302, 187–194. 10.1016/j.ijmm.2012.07.007 22939033

[B34] Joshi B. Biswas S. Sharma Y. D. (1992). Effect of Heat-Shock on Plasmodium Falciparum Viability, Growth and Expression of the Heat-Shock Protein 'PFHSP70-I' Gene. FEBS Lett. 312, 91–94. 10.1016/0014-5793(92)81417-k 1385215

[B35] Kadioglu O. Chan A. Cong Ling Qiu A. Wong V. K. W. Colligs V. Wecklein S. (2017). Artemisinin Derivatives Target Topoisomerase 1 and Cause DNA Damage In Silico and *In Vitro* . Front. Pharmacol. 8, 711. 10.3389/fphar.2017.00711 29062278 PMC5640709

[B36] Kafsack B. F. C. Llinás M. (2010). Eating at the Table of Another: Metabolomics of Host-Parasite Interactions. Cell Host & Microbe 7, 90–99. 10.1016/j.chom.2010.01.008 20159614 PMC2825149

[B37] Kehr S. Sturm N. Rahlfs S. Przyborski J. M. Becker K. (2010). Compartmentation of Redox Metabolism in Malaria Parasites. Plos Pathog. 6, e1001242. 10.1371/journal.ppat.1001242 21203490 PMC3009606

[B38] Lang-Unnasch N. Murphy A. D. (1998). Metabolic Changes of the Malaria Parasite during the Transition from the Human to the Mosquito Host. Annu. Rev. Microbiol. 52, 561–590. 10.1146/annurev.micro.52.1.561 9891808

[B39] Le Bras J. Durand R. pharmacology c. (2003). The Mechanisms of Resistance to Antimalarial Drugs in Plasmodium Falciparum. Fundam. Clin. Pharmacol. 17, 147–153. 10.1046/j.1472-8206.2003.00164.x 12667224

[B40] MalariaGEN Plasmodium falciparum Community Project (2016). Genomic Epidemiology of Artemisinin Resistant Malaria. Elife 5, e08714. 10.7554/eLife.08714 26943619 PMC4786412

[B41] Mbengue A. Bhattacharjee S. Pandharkar T. Liu H. Estiu G. Stahelin R. V. (2015). A Molecular Mechanism of Artemisinin Resistance in Plasmodium Falciparum Malaria. Nature 520, 683–687. 10.1038/nature14412 25874676 PMC4417027

[B42] Ménard D. Khim N. Beghain J. Adegnika A. A. Shafiul-Alam M. Amodu O. (2016). A Worldwide Map ofPlasmodium falciparumK13-Propeller Polymorphisms. N. Engl. J. Med. 374, 2453–2464. 10.1056/nejmoa1513137 27332904 PMC4955562

[B43] Miotto O. Amato R. Ashley E. A. MacInnis B. Almagro-Garcia J. Amaratunga C. (2015). Genetic Architecture of Artemisinin-Resistant Plasmodium Falciparum. Nat. Genet. 47, 226–234. 10.1038/ng.3189 25599401 PMC4545236

[B44] Modrzynska K. Pfander C. Chappell L. Yu L. Suarez C. Dundas K. (2017). A Knockout Screen of ApiAP2 Genes Reveals Networks of Interacting Transcriptional Regulators Controlling the Plasmodium Life Cycle. Cell Host & Microbe 21, 11–22. 10.1016/j.chom.2016.12.003 28081440 PMC5241200

[B45] Mok S. Imwong M. Mackinnon M. J. Sim J. Ramadoss R. Yi P. (2011). Artemisinin Resistance in Plasmodium Falciparum Is Associated with an Altered Temporal Pattern of Transcription. BMC Genomics 12, 391–414. 10.1186/1471-2164-12-391 21810278 PMC3163569

[B46] Mok S. Ashley E. A. Ferreira P. E. Zhu L. Lin Z. Yeo T. (2015). Population Transcriptomics of Human Malaria Parasites Reveals the Mechanism of Artemisinin Resistance. Science 347, 431–435. 10.1126/science.1260403 25502316 PMC5642863

[B47] Mok S. Stokes B. H. Gnädig N. F. Ross L. S. Yeo T. Amaratunga C. (2021). Artemisinin-resistant K13 Mutations Rewire Plasmodium Falciparum’s Intra-erythrocytic Metabolic Program to Enhance Survival. Nat. Commun. 12, 1–15. 10.1038/s41467-020-20805-w 33483501 PMC7822823

[B48] Mundwiler-Pachlatko E. Beck H.-P. (2013). Maurer's Clefts, the enigma of Plasmodium Falciparum. Proc. Natl. Acad. Sci. 110, 19987–19994. 10.1073/pnas.1309247110 24284172 PMC3864307

[B49] Nair S. Li X. Arya G. A. McDew-White M. Ferrari M. Nosten F. (2018). Fitness Costs and the Rapid Spread of Kelch13-C580y Substitutions Conferring Artemisinin Resistance. Antimicrob. Agents Chemother. 62, e00605–18. 10.1128/AAC.00605-18 29914963 PMC6125530

[B50] Nepveu F. Turrini F. (2013). Targeting the Redox Metabolism of Plasmodium Falciparum. Future Med. Chem. 5, 1993–2006. 10.4155/fmc.13.159 24175748

[B51] Olszewski K. L. Morrisey J. M. Wilinski D. Burns J. M. Vaidya A. B. Rabinowitz J. D. (2009). Host-parasite Interactions Revealed by Plasmodium Falciparum Metabolomics. Cell Host & Microbe 5, 191–199. 10.1016/j.chom.2009.01.004 19218089 PMC2737466

[B52] Painter H. J. Campbell T. L. Llinás M. parasitology b. (2011). The Apicomplexan AP2 Family: Integral Factors Regulating Plasmodium Development. Mol. Biochem. Parasitol. 176, 1–7. 10.1016/j.molbiopara.2010.11.014 21126543 PMC3026892

[B53] Prajapati S. K. Singh O. P. (2013). Remodeling of Human Red Cells Infected with Plasmodium Falciparum and the Impact of PHIST Proteins. Blood Cell Mol. Dis. 51, 195–202. 10.1016/j.bcmd.2013.06.003 23880461

[B54] Prieto J. H. Koncarevic S. Park S. K. Yates J. III Becker K. (2008). Large-Scale Differential Proteome Analysis in Plasmodium Falciparum under Drug Treatment. PLoS ONE 3, e4098. 10.1371/journal.pone.0004098 19116658 PMC2605551

[B55] Rathod P. K. McErlean T. Lee P.-C. (1997). Variations in Frequencies of Drug Resistance in Plasmodium Falciparum. Proc. Natl. Acad. Sci. 94, 9389–9393. 10.1073/pnas.94.17.9389 9256492 PMC23200

[B56] Rawat M. Kanyal A. Sahasrabudhe A. Vembar S. S. Lopez-Rubio J.-J. Karmodiya K. J. S. r. (2021). Histone Acetyltransferase PfGCN5 Regulates Stress Responsive and Artemisinin Resistance Related Genes in Plasmodium Falciparum. Scientific Rep. 11, 1–13. 10.1038/s41598-020-79539-w PMC7806804 33441725

[B57] Rocamora F. Zhu L. Liong K. Y. Dondorp A. Miotto O. Mok S. (2018). Oxidative Stress and Protein Damage Responses Mediate Artemisinin Resistance in Malaria Parasites. Plos Pathog. 14, e1006930. 10.1371/journal.ppat.1006930 29538461 PMC5868857

[B58] Santos J. M. Josling G. Ross P. Joshi P. Orchard L. Campbell T. (2017). Red Blood Cell Invasion by the Malaria Parasite Is Coordinated by the PfAP2-I Transcription Factor. Cell Host & Microbe 21, 731–741. e10. 10.1016/j.chom.2017.05.006 28618269 PMC5855115

[B59] Schulze J. Kwiatkowski M. Borner J. Schlüter H. Bruchhaus I. Burmester T. (2015). ThePlasmodium Falciparumexportome Contains Non-canonical PEXEL/HT Proteins. Mol. Microbiol. 97, 301–314. 10.1111/mmi.13024 25850860

[B60] Siddiqui F. A. Cabrera M. Wang M. Brashear A. Kemirembe K. Wang Z. (2018). Plasmodium Falciparum Falcipain-2a Polymorphisms in Southeast Asia and Their Association with Artemisinin Resistance. J. Infect. Dis. 218, 434–442. 10.1093/infdis/jiy188 29659945 PMC6048984

[B61] Siddiqui G. Srivastava A. Russell A. S. Creek D. J. (2017). Multi-omics Based Identification of Specific Biochemical Changes Associated with PfKelch13-Mutant Artemisinin-Resistant Plasmodium Falciparum. J. Infect. Dis. 215, 1435–1444. 10.1093/infdis/jix156 28368494

[B62] Slater H. C. Griffin J. T. Ghani A. C. Okell L. C. (2016). Assessing the Potential Impact of Artemisinin and Partner Drug Resistance in Sub-saharan Africa. Malar. J. 15, 1–11. 10.1186/s12936-015-1075-7 26739092 PMC4704433

[B63] Spielmann T. Gras S. Sabitzki R. Meissner M. (2020). Endocytosis in Plasmodium and Toxoplasma Parasites. Trends Parasitol. 36, 520–532. 10.1016/j.pt.2020.03.010 32340866

[B64] Srivastava A. Philip N. Hughes K. R. Georgiou K. MacRae J. I. Barrett M. P. (2016). Stage-specific Changes in Plasmodium Metabolism Required for Differentiation and Adaptation to Different Host and Vector Environments. Plos Pathog. 12, e1006094. 10.1371/journal.ppat.1006094 28027318 PMC5189940

[B65] Stokes B. H. Rubiano K. Dhingra S. K. Mok S. Straimer J. Gnädig N. F. (2021). Plasmodium Falciparum K13 Mutations in Africa and Asia Present Varying Degrees of Artemisinin Resistance and an Elevated Fitness Cost in African Parasites. Elife 10, e66277. 10.7554/eLife.66277 34279219 PMC8321553

[B66] Suresh N. Haldar K. (2018). Mechanisms of Artemisinin Resistance in Plasmodium Falciparum Malaria. Curr. Opin. Pharmacol. 42, 46–54. 10.1016/j.coph.2018.06.003 30077118 PMC6314025

[B67] Swift M. L. sciences c. (1997). GraphPad Prism, Data Analysis, and Scientific Graphing. J. Chem. Inf. Comput. Sci. 37, 411–412. 10.1021/ci960402j

[B68] Teuscher F. Gatton M. L. Chen N. Peters J. Kyle D. E. Cheng Q. (2010). Artemisinin‐Induced Dormancy inPlasmodium Falciparum: Duration, Recovery Rates, and Implications in Treatment Failure. J. Infect. Dis. 202, 1362–1368. 10.1086/656476 20863228 PMC2949454

[B69] Tewari S. G. Swift R. P. Reifman J. Prigge S. T. Wallqvist A. (2020). Metabolic Alterations in the Erythrocyte during Blood-Stage Development of the Malaria Parasite. Malar. J. 19, 94–18. 10.1186/s12936-020-03174-z 32103749 PMC7045481

[B70] Thélu J. Burnod J. Bracchi V. Ambroise-Thomas P. biology c. (1994). Identification of Differentially Transcribed RNA and DNA Helicase-Related Genes ofPlasmodium Falciparum. DNA Cell Biol. 13, 1109–1115. 10.1089/dna.1994.13.1109 7702753

[B71] Tippmann S. (2015). Programming Tools: Adventures with R. Nature 517, 109–110. 10.1038/517109a 25557714

[B72] Tirrell A. R. Vendrely K. M. Checkley L. A. Davis S. Z. McDew-White M. Cheeseman I. H. (2019). Pairwise Growth Competitions Identify Relative Fitness Relationships Among Artemisinin Resistant Plasmodium Falciparum Field Isolates. Malar. J. 18, 295–313. 10.1186/s12936-019-2934-4 31462253 PMC6714446

[B73] Tougan T. Edula J. R. Morita M. Takashima E. Honma H. Tsuboi T. (2020). The Malaria Parasite Plasmodium Falciparum in Red Blood Cells Selectively Takes up Serum Proteins that Affect Host Pathogenicity. Malar. J. 19, 155–213. 10.1186/s12936-020-03229-1 32295584 PMC7161009

[B74] Tuteja R. Ansari A. Chauhan V. S. (2011). Emerging Functions of Transcription Factors in Malaria Parasite. J. Biomed. Biotechnol. 2011, 461979. 10.1155/2011/461979 22131806 PMC3216465

[B75] Tuteja R. (2010). Genome Wide Identification of Plasmodium Falciparum Helicases: a Comparison with Human Host. Cell Cycle 9, 104–120. 10.4161/cc.9.1.10241 20016272

[B76] Tuteja R. Pradhan A. (2006). Unraveling the 'DEAD-Box' Helicases of Plasmodium Falciparum. Gene 376, 1–12. 10.1016/j.gene.2006.03.007 16713133 PMC7127577

[B77] Uwimana A. Legrand E. Stokes B. H. Ndikumana J.-L. M. Warsame M. Umulisa N. (2020). Emergence and Clonal Expansion of *In Vitro* Artemisinin-Resistant Plasmodium Falciparum Kelch13 R561H Mutant Parasites in Rwanda. Nat. Med. 26, 1602–1608. 10.1038/s41591-020-1005-2 32747827 PMC7541349

[B78] Van Biljon R. Van Wyk R. Painter H. J. Orchard L. Reader J. Niemand J. (2019). Hierarchical Transcriptional Control Regulates Plasmodium Falciparum Sexual Differentiation. BMC Genomics 20, 920–1016. 10.1186/s12864-019-6322-9 31795940 PMC6889441

[B79] Venkatesan M. Gadalla N. B. Stepniewska K. Dahal P. Nsanzabana C. Moriera C. (2014). Polymorphisms in Plasmodium Falciparum Chloroquine Resistance Transporter and Multidrug Resistance 1 Genes: Parasite Risk Factors that Affect Treatment Outcomes for P. Falciparum Malaria after Artemether-Lumefantrine and Artesunate-Amodiaquine. falciparum Malar. after artemether-lumefantrine artesunate-amodiaquine 91, 833–843. 10.4269/ajtmh.14-0031 PMC4183414 25048375

[B80] Wang S. Xu S. Geng J. Si Y. Zhao H. Li X. (2020). Molecular Surveillance and *In Vitro* Drug Sensitivity Study of Plasmodium Falciparum Isolates from the China-Myanmar Border. Am. J. Trop. Med. Hyg. 103, 1100–1106. 10.4269/ajtmh.20-0235 32588794 PMC7470591

[B81] Wang Z. Cabrera M. Yang J. Yuan L. Gupta B. Liang X. (2016). Genome-wide Association Analysis Identifies Genetic Loci Associated with Resistance to Multiple Antimalarials in Plasmodium Falciparum from China-Myanmar Border. Sci. Rep. 6, 33891–33912. 10.1038/srep33891 27694982 PMC5046179

[B82] Warncke J. D. Beck H.-P. J. M. Reviews M. B. (2019). Host Cytoskeleton Remodeling throughout the Blood Stages of Plasmodium Falciparum. Microbiol. Mol. Biol. Rev. MMBR 83. 10.1128/mmbr.00013-19 PMC6759665 31484690

[B83] Warncke J. D. Vakonakis I. Beck H.-P. Reviews M. B. (2016). Plasmodium Helical Interspersed Subtelomeric (PHIST) Proteins, at the center of Host Cell Remodeling. Microbiol. Mol. Biol. Rev. 80, 905–927. 10.1128/mmbr.00014-16 27582258 PMC5116875

[B84] Wilairat P. Kümpornsin K. Chookajorn T. (2016). Plasmodium Falciparum Malaria: Convergent Evolutionary Trajectories towards Delayed Clearance Following Artemisinin Treatment. Med. Hypotheses 90, 19–22. 10.1016/j.mehy.2016.02.022 27063079

[B85] Witkowski B. Amaratunga C. Khim N. Sreng S. Chim P. Kim S. (2013). Novel Phenotypic Assays for the Detection of Artemisinin-Resistant Plasmodium Falciparum Malaria in Cambodia: *In-Vitro* and *Ex-Vivo* Drug-Response Studies. Lancet Infect. Dis. 13, 1043–1049. 10.1016/s1473-3099(13)70252-4 24035558 PMC5015432

[B86] World Health Organization (2020). World Malaria Report 2020: 20 Years of Global Progress and Challenges. Geneva, Switzerland: World Health Organization.

[B87] Yang T. Yeoh L. M. Tutor M. V. Dixon M. W. McMillan P. J. Xie S. C. (2019). Decreased K13 Abundance Reduces Hemoglobin Catabolism and Proteotoxic Stress, Underpinning Artemisinin Resistance. Cell Rep. 29, 2917–2928. e5. 10.1016/j.celrep.2019.10.095 31775055

[B88] Zuzarte-Luís V. Mota M. M. (2018). Parasite Sensing of Host Nutrients and Environmental Cues. Cell Host & Microbe 23, 749–758. 10.1016/j.chom.2018.05.018 29902440

