# Supplementary material for: Identification of Co-Existing Mutations and Gene Expression Trends Associated With K13-Mediated Artemisinin Resistance in Plasmodium falciparum
Source: Front Genet. 2022 Apr 6;13:824483. doi: 10.3389/fgene.2022.824483 (PMC9019836; doi:10.3389/fgene.2022.824483)
Supplement: Supplementary file 1 [file DataSheet1.zip › Data_files/Supplementary Information.docx]

**Supplementary Material**

**Supplementary Figure 1: Coexistence of different SNPs along with K13 mutations.** Bar graph showing the number of SNP present over different genes in different chromosome of *P. falciparum*.

**
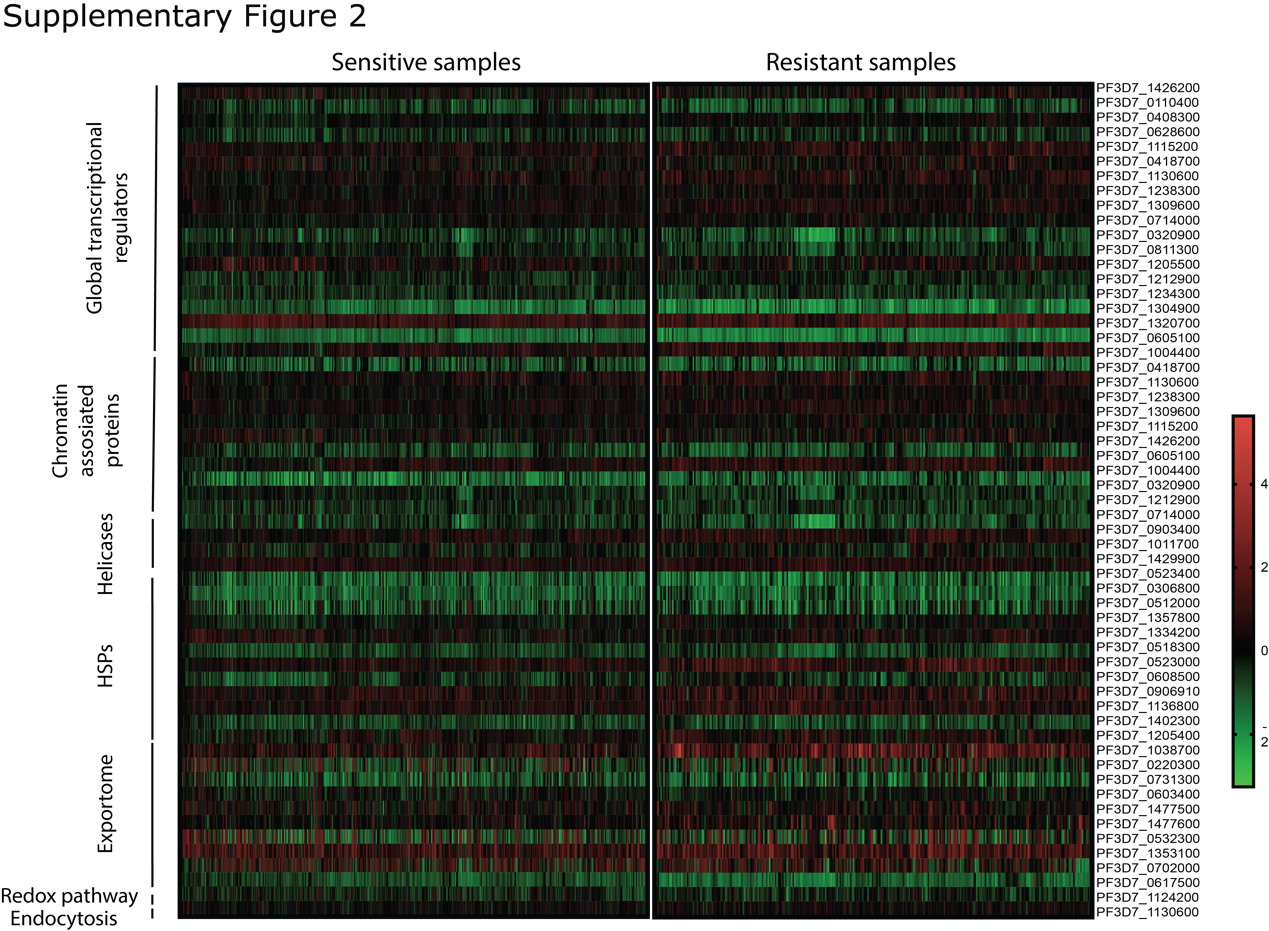
**

**Supplementary Figure 2: Artemisinin resistance transcriptome of different biological pathways.** Transcriptomics data from Mok *et al.,* 2015 study were used to plot this heat map. Samples with parasite clearance half-life less than 3h were considered as sensitive and those with clearance half-life more than 5h were considered as resistance. Genes deregulated in Mok *et al.,* 2015 study and common with either Mok *et al.,* 2011 or Rocomora *et al.,* 2018 were plotted. Heat map were generated using GraphPad (http://www.graphpad.com/faq/viewfaq.cfm?faq=1362).

**Supplementary Table legends**

**Supplementary Table 1:** Table depicting the frequencies of occurrence of WT or mutant version of K13 with WT or mutant of individual candidate background genes in a 2X2 matrix used for the chi-squared test. 10 background gene candidates and their mutant/WT counterparts were tested in the analysis. All except Pfmdr1 mutants turned up as significantly correlated with K13 mutations.

**Supplementary Table 2:** The criteria used in studies (Mok et al 2011, Mok et al 2015 and Rocomora 2018) to define significantly deregulated genes.

**Supplementary Table 3:** Table showing the presence (1) and absence (0) of different K13 mutation in different isolates. Isolates showing more than one K13 mutations are highlighted, Blue color (South east Asia) and Green color (African region). It also shows the percentage of K13 mutations identified in different countries.

**Supplementary Table 4:** Table showing the mutations co-existing along with the K13 mutations. It represents SNPs which were identified in 75% of the artemisinin resistant isolates (having K13 mutation and known to enhance resistance in literature) and less than 25% of the sensitive parasites used for the analysis.

**Supplementary Table 5:** Table showing the different classes of genes which are used for transcriptomics analysis along with their deregulation in different studies. Transcriptome data from Mok *et al*., 2011, Mok *et al.,* 2015 and Rocomora *et al.,* 2018 data have been represented in the Table. Genes which were significantly upregulated were shown by red color and those which are down regulated are shown by blue color.
